# Supplementary material for: The causal role of affect sharing in driving vicarious fear learning
Source: PLoS One. 2022 Nov 18;17(11):e0277793. doi: 10.1371/journal.pone.0277793 (PMC9674158; doi:10.1371/journal.pone.0277793)
Supplement: S1 File — (DOCX) [file pone.0277793.s001.docx]

**Supplementary Methods**

**Vicarious fear conditioning paradigm**

*Learning stage: videos*

We used only male demonstrators in the videos. Anecdotal evidence suggests that gender match between demonstrator and participant has no effect (Andreas Olsson, personal communication), while responses differ to watching a man as opposed to a woman in pain (Gulas, McKeage, & Weinberger, 2010), irrespective of the participant’s gender.

*Instructions*

Upon signing the informed consent form, participants were informed that they might receive “unpleasant but not painful electric shocks delivered by a shock electrode attached to the right hand” during the hypnosis session.

To avoid participants becoming self-conscious about their eye movements, they were told that pupil diameter, in addition to skin conductance level would be recorded as measures of their level of alertness.

After placing all electrodes, participants received the following instruction:

“Once you have reached the hypnotic state, you will see videos of another person completing the same experiment that you will afterwards complete yourself. Just like you, the person in the videos is wearing a shock electrode and is looking at a monitor. Different colored squares will appear on the person’s monitor. Following some of the colored squares, the person will receive an electric shock. Please watch the videos as attentively as possible, paying attention to both the person and the squares. During this first part of the session, you will not receive any shocks yourself. Afterwards, you will complete the same experiment as the person in the videos. In this second part of the session, you may receive electric shocks. You will receive between 0 and 4 shocks delivered via the shock electrode attached to your right hand (pointing at it). The shocks may be unpleasant, but will be bearable. You will see reminders of these instructions later on your monitor. Before these two parts, I will guide you into a hypnotic state and will administer a suggestion to you. We will repeat the two parts afterwards with another suggestion. At the end I will guide you back out of the hypnotic state”.

Directly before each learning stage, the following reminder appeared on screen:

“You will now see videos of another person completing the same experiment that you will afterwards complete yourself. Different colored squares will appear on the person’s monitor. Following some of the colored squares, the person will receive an electric shock. Please pay attention to both the person and the squares.”

Directly before each test stage, the following reminder appeared on screen:

“You will now complete the same experiment as the person in the videos. You may receive between 0 and 4 electric shocks. Please pay attention to the squares.”

*Hypnotic suggestion for high affect sharing*

“...We will get to the task in a moment, but for now, listen to me carefully: Our personality or our self consists of different parts… or aspects or sides of ourselves… sometimes, we are shy and sometimes confident, sometimes afraid and other times surprisingly courageous… Your self also includes different parts, existing in you, side by side, completing each other…

I am now going to address one of these sides of your self, the one particularly open for others‘ emotional feelings… This part is especially sensitive to how other people are feeling. Like a parent who has this fine intuition for their child, there is a part of you with fine antennae for another person´s emotions… Think of a situation or a person, where this side of you shows. Maybe there is a beloved person, a person really important to you… You know the feeling of being especially open for this person´s emotions. You have the ability to share this person‘s feelings, to feel what they feel… And even though they are another person, you can feel more distinctively what they feel…. Maybe it is like an open window, letting the emotions flow freely between two persons… You can allow the other person´s sensations to reach you… Take your time. As soon as you are ready and this part of you is really present, inform me by a nod… (wait for nod) Very good, some people describe a part or a side of themselves as a person, as an image or as a feeling… now please think about a name for this part of you and when you are ready, tell me this name… (wait for name).

Welcome, (name), you are a part of (participant‘s name) and you are particularly open for other people‘s emotions. Think about what makes you notice that you are particularly able to perceive other persons‘ feelings so very sensitively. For this, you possess a magical inner dial, and by this dial you can increase or decrease the incoming sensations… when you increase, your openness for others‘ emotions rises… Increase your own openness as far up as you can go… you are now going to see video clips… you do not know the person in the videos… it is an unfamiliar person and yet you will feel connected to this person in a magical way… while you watch the person attentively, you will notice that you are surprisingly open to their emotions… but only as far as you can cope with in this state of openness. Just be curious about how you will experience the video clips… (name), now open your eyes and be curious about what is coming… Pay attention to both the person and the squares in the video…”.

*Hypnotic suggestion for low affect sharing*

“Soon, I will address another part of your self, a part that is particularly closed against others' emotional feelings… little by little, you can remember situations in which you were particularly closed... In these situations you may have consciously decided to keep a distance to other person's feelings… there are situations when it is wiser to close oneself up… to seal off other person's emotions… some people experience this reserve, this feeling of being closed, while watching movies, consciously deciding not to engage in the emotions presented in the movie in order to protect themselves… You can think of your very own, personal experiences… closed… closed like a firmly locked window, which keeps everything unwelcome outside… enclosed like behind robust bulletproof glass… think about how you feel in this situation… how you do not feel actually… take your time. When you are ready, when this side of you is fully present, nod briefly to give me a sign…. (wait for nod) very good… now think of a name for this part or this side of you. Tell me the name when you are ready… (wait for name)

Welcome, (name), you are a part of (participant‘s name) and you are particularly closed up against other person's emotions. Think about what makes you notice that you can hardly or even not at all perceive others' feelings... Now we get to the video clips again. In a moment you will watch videos… you do not know the person in the clips… it is an unfamiliar person and you will feel a large distance to them… even though you can clearly see the person, you will feel completely separated from their sensations, closed and separated, like from behind strong bulletproof glass… While watching the person closely throughout the video, you will notice how closed you will be against their emotions… just be curious about how you will experience the video clips… (name), open your eyes now and be curious about what is coming…Pay attention to both the person and the squares in the video…”.

*Physiological measures*

Electrocardiogram (ECG, outside scope of present paper), skin conductance and eye movements were recorded throughout the paradigm. Eye movements of the participant’s dominant eye were recorded with an EyeLink 1000 Plus Desktop Mount Eye Tracker (SR Research Ltd., Kanata, Ontario, Canada). Each participant was instructed that they could move their gaze freely on the computer screen, but not beyond, as this would make measurements impossible.

*Post-hypnosis interview*

Upon completion of the hypnosis session, participants immediately answered a series of questions presented on screen, first for the second learning and test stage (while still fresh in their memory), then for the first learning and test stage. Participants were shown pictures of the demonstrator and the two colored rectangles used with that demonstrator. They estimated how many electric shocks (1) the demonstrator (during the learning stage) / (2) they themselves (during the test stage) had received with each color, using a 7-point numerical rating scale ranging from 0 (none) to 6+ (6 or more). They rated (3) how unpleasant the shocks were for the demonstrator and (4) how unpleasant it was for them to watch these videos, (6) how likeable they found the demonstrator, (9) how hypnotized they felt during each of the 4 stages and (10) how effective the first / second suggestion was, using an 11-point numerical rating scale ranging from 0 (not at all) to 10 (extremely so), respectively. They also indicated (5) whether or not they had felt any tingling and / or pain in their own body while watching the demonstrator receive electric shocks (yes/no response), (7) whether or not, after seeing the first and second video series, respectively, they had expected that they themselves would now receive electric shocks (rather yes / rather no), and (8) how intense they had imagined those shocks to be (0-10 scale). Participants also answered the following open-ended questions, separately for each suggestion: (11) What strategy did you use to implement the suggestion (e.g. what did you imagine / remember / perceive)? (12) What changes did the suggestion induce while you watched the video (e.g. regarding how you felt yourself and/or how you experienced the video)? All answers were given orally and were recorded and transcribed by the experimenter.

**Self-report measures of empathy and personality**

To characterize the sample, which was pre-screened for high hypnotic suggestibility, we collected several personality measures. After a 15-min break following the hypnosis session, questionnaires were administered on screen to assess trait empathy (Questionnaire of Cognitive and Affective Empathy – QCAE; Georgi, Petermann, & Schipper, 2014) and the tendency to experience vicarious pain (using an ad-hoc German translation of the vicarious pain task by Osborn & Derbyshire, 2010). Autistic traits were assessed using the German version of the Autism Quotient (Baron-Cohen, Wheelwright, Skinner, Martin, & Clubley, 2001), and trait anxiety was assessed with the State-Trait Anxiety Inventory-Form X2 (STAI-X2; Laux, Glanzmann, Schaffner, & Spielberger, 1981). State anxiety was assessed at the very beginning of the experimental session (STAI-X1 Short Form; Grimm, 2009). Average results of the trait measures are shown in Table G.

**Data analysis**

All scripts used for the preprocessing, statistical analysis and plotting of the data of this study, as well as an analysis walk-through, are available as part of the Supplementary Material.

**Supplementary Results**

Qualitative results of the post-hypnotic interview on strategies used and effects obtained from the hypnotic suggestions are shown in Table A. Complete results of all ANOVA models assessing the effect of hypnotic suggestions for high versus low affect sharing on self-report (Table B), skin conductance response (SCR; Table C), tonic skin conductance level (SCL; Table D) and eye gaze (Table E) are shown in the Supplementary Tables B-E. The effect of hypnotic suggestions on contingency memory accuracy, calculated as the absolute difference between actual and remembered number of shocks delivered to the demonstrator, is depicted in Figure A. ANOVA results for SCR and SCL calculated separately for each round and compared between rounds are shown in Table F. Effects of the hypnotic suggestions on self-report (Figure B) and on SCR/SCL (Figure D) shown separately for the two groups receiving the high affect sharing condition in round 1 or 2 are displayed in Figure B and Figure D. Average results of our participants on the assessed trait measures of empathy and personality are shown in Table G. Spearman correlations between SCR , SCL and eye gaze indices are displayed in Table H-J. The individual participants’ unpleasantness ratings (self-report) as well as unconditioned responses (SCR) to the demonstrator’s pain observed during learning under high versus low affect sharing are shown in Figure C and Figure E. The raw data collected in this study are available as part of the Supplemental Material in the file “00rawdata.sav” (explanations on the variable meanings are given in the file “00variable_labels.xlsx”).

**Table A:**

*Results of the post-hypnosis interview regarding strategies used by the participants to implement the hypnotic suggestions and changes (effects) induced by the suggestions while participants watched the video, respectively (percentage of participants mentioning each category as strategy and/or effect is shown).*

| Mental activity mentioned as strategy and/or effect of the suggestion: | Hypnotic suggestion: | | | |
| --- | --- | --- | --- | --- |
|  | High affect sharing | | Low affect sharing | |
|  | Strategy^a^: | Effect^b^: | Strategy^a^: | Effect^b^: |
| 1. Scenes: Remember or imagine scenes in which I felt emotionally open vs. closed towards someone (e.g. “I imagined walking by beggars in the street and feeling annoyed with them”) | 62% | 0% | 67% | 0% |
| 1. Relationship: Imagine demonstrator as loved vs. disliked person (e.g. “I imagined my boyfriend”)* | 49% | 18% | 15% | 15% |
| 1. Symbol: Imagine a picture symbolizing how I feel (e.g. “I was hard like a stone”) | 0% | 3% | 18% | 3% |
| 1. Affect sharing / caring vs. no affect sharing / feeling distant / not caring (e.g. “I was worried for him”, “I felt his pain in my own body”) | 5% | 64% | 23% | 64% |
| 1. Mentalizing vs. not mentalizing (e.g. “I tried to imagine how he felt”) | 10% | 26% | 3% | 10% |
| 1. Focus attention towards vs. away from emotional cues (e.g. “I paid more attention to his face”)** | 8% | 21% | 5% | 8% |
| 1. Intensity: Reappraise perceived intensity of the demonstrator’s suffering (e.g. “I imagined that he was just acting, similar to when I watch horror movies”)** | 0% | 0% | 15% | 8% |
| 1. Responsibility: reappraise demonstrator as being vs. not being responsible for his own suffering (“I reminded myself that he had agreed to doing this”)** | 0% | 0% | 3% | 0% |
| 1. “Schadenfreude” (“I felt malicious joy”)** | 0% | 0% | 0% | 3% |
| 1. Other strategy / outcome (e.g. “I was afraid of him”, “I felt morose”) | 0% | 5% | 5% | 13% |
| 1. No response | 5% | 3% | 3% | 8% |

*Note*. Responses to the open-ended questions ^a^)„What strategy did you use to implement the suggestion?“ and ^b^) „What changes did the suggestion induce while you watched the video?“ were sortet post-hoc into 10 categories.

*) related to the “deservedness” category described in Zaki (2014).

**) as described in Zaki (2014).

**Table B:**

*Results of Mixed Analysis of Variance (ANOVA) Models Used to Explain Self-Report in N = 39 Participants*

| ANOVA model | Effect | *df_hypothesis_* | *df_error_* | *F* | *p* | _partial_ ƞ^2^ [90% CI] |
| --- | --- | --- | --- | --- | --- | --- |
| *suggestion x target x order => unpleasantness* | | |  |  |  |  |
|  | target (self vs. demonstrator) | 1 | 37 | 53.39 | <.001 | .59 [.40, .69] |
|  | target x order | 1 | 37 | 0.90 | .349 | .02 [.00, .15] |
|  | suggestion | 1 | 37 | 67.72 | <.001 | .65 [.47, .74] |
|  | suggestion x order | 1 | 37 | 4.34 | .044 | .11 [.002, .27] |
|  | target x suggestion | 1 | 37 | 39.28 | <.001 | .52 [.31, .64] |
|  | target x suggestion x order | 1 | 37 | 3.92 | .055 | .10 [.00, .26] |
|  | order | 1 | 37 | 1.94 | .172 | .05 [.00, .19] |
| *suggestion x order => sympathy* | |  |  |  |  |  |
|  | suggestion | 1 | 37 | 38.71 | <.001 | .51 [.31, .63] |
|  | suggestion x order | 1 | 37 | 0.13 | .721 | .00 [.00, .08] |
|  | order | 1 | 37 | 2.70 | .109 | .07 [.00, .22] |
| *suggestion x order => efficacy of the suggestion* | | | | |  |  |
|  | suggestion | 1 | 37 | 0.85 | .363 | .02 [.00, .14] |
|  | suggestion x order | 1 | 37 | 0.98 | .328 | .03 [.00, .15] |
|  | order | 1 | 37 | 1.11 | .298 | .03 [.00, .16] |
| *suggestion x CS x order => estimated no. of shocks to demonstrator* | | | |  |  |  |
|  | suggestion | 1 | 37 | 1.21 | .279 | .03 [.00, .16] |
|  | suggestion x order | 1 | 37 | 8.50 | .006 | .19 [.03, .36] |
|  | CS | 1 | 37 | 62.49 | <.001 | .63 [.45, .72] |
|  | CS x order | 1 | 37 | 0.90 | .350 | .02 [.00, .15] |
|  | suggestion x CS | 1 | 37 | 0.65 | .427 | .02 [.00, .13] |
|  | suggestion x CS x order | 1 | 37 | 0.65 | .427 | .02 [.00, .13] |
|  | order | 1 | 37 | 0.49 | .487 | .01 [.00, .12] |
| *suggestion x CS x order => absolute error in estimated no. of shocks to demonstrator* | | | | | |  |
|  | suggestion | 1 | 37 | 0.47 | .498 | .01 [.00, .12] |
|  | suggestion x order | 1 | 37 | 0.26 | .617 | .01 [.00, .10] |
|  | CS | 1 | 37 | 33.28 | <.001 | .47 [.27, .60] |
|  | CS x order | 1 | 37 | 1.50 | .228 | .04 [.00, .18] |
|  | suggestion x CS | 1 | 37 | 1.47 | .234 | .04 [.00, .17] |
|  | suggestion x CS x order | 1 | 37 | 3.58 | .066 | .09 [.00, .25] |
|  | order | 1 | 37 | 1.83 | .184 | .05 [.00, .19] |

*Note.* CS – conditioned stimulus.

**Table C:**

*Results of Mixed Analysis of Variance (ANOVA) Models Used to Explain Skin Conductance Response (SCR) in N = 39 Participants.*

| ANOVA model | Effect | *df_hypothesis_* | *df_error_* | *F* | *p* | _partial_ ƞ^2^ [90% CI] |
| --- | --- | --- | --- | --- | --- | --- |
| *suggestion x CS x order => SCR to CS (learning stage)* | | |  |  |  |  |
|  | suggestion | 1 | 37 | 0.08 | .774 | .00 [.00, .07] |
|  | suggestion x order | 1 | 37 | 13.25 | .001 | .26 [.08, .43] |
|  | CS | 1 | 37 | 1.55 | .221 | .04 [.00, .18] |
|  | CS x order | 1 | 37 | 0.73 | .397 | .02 [.00, .14] |
|  | suggestion x CS | 1 | 37 | 0.08 | .781 | .00 [.00, .07] |
|  | suggestion x CS x order | 1 | 37 | 0.42 | .520 | .01 [.00, .12] |
|  | order | 1 | 37 | 1.41 | .243 | .04 [.00, .17] |
| *suggestion x US x order => SCR to US (learning stage)* | | |  |  |  |  |
|  | suggestion | 1 | 37 | 10.72 | .002 | .23 [.05, .39] |
|  | suggestion x order | 1 | 37 | 19.94 | <.001 | .35 [.15, .50] |
|  | US | 1 | 37 | 65.33 | <.001 | .64 [.46, .73] |
|  | US x order | 1 | 37 | 4.36 | .044 | .11 [.002, .27] |
|  | suggestion x US | 1 | 37 | 12.40 | .001 | .25 [.07, .42] |
|  | suggestion x US x order | 1 | 37 | 9.81 | .003 | .21 [.05, .38] |
|  | order | 1 | 37 | 5.04 | .031 | .12 [.01, .28] |
| *suggestion x CS x order => SCR to CS (test stage)* | | |  |  |  |  |
|  | suggestion | 1 | 37 | 1.73 | .197 | .04 [.00, .18] |
|  | suggestion x order | 1 | 37 | 63.77 | <.001 | .63 [.46, .73] |
|  | CS | 1 | 37 | 21.26 | <.001 | .36 [.16, .52] |
|  | CS x order | 1 | 37 | 0.09 | .766 | .00 [.00, .08] |
|  | suggestion x CS | 1 | 37 | 5.68 | .022 | .13 [.01, .30] |
|  | suggestion x CS x order | 1 | 37 | 0.39 | .535 | .01 [.00, .11] |
|  | order | 1 | 37 | 1.40 | .244 | .04 [.00, .17] |

*Note.* CS – conditioned stimulus; US – unconditioned stimulus.

**Table D:**

*Results of a Mixed Analysis of Variance (ANOVA) Model Used to Explain Tonic Skin Conductance Level (SCL) in N = 39 Participants*

| ANOVA model | Effect | *df_hypothesis_* | *df_error_* | *F* | *p* | _partial_ ƞ^2^ [90% CI] |
| --- | --- | --- | --- | --- | --- | --- |
| *Stage (learning vs. test) x suggestion x order => SCL* | | | | | | |
|  | stage | 1 | 37 | 0.15 | .699 | .00 [.00, .09] |
|  | stage x order | 1 | 37 | 0.96 | .334 | .03 [.00, .15] |
|  | suggestion | 1 | 37 | 0.71 | .405 | .02 [.00, .14] |
|  | suggestion x order | 1 | 37 | 32.48 | <.001 | .47 [.26, .60] |
|  | stage x suggestion | 1 | 37 | 6.29 | .017 | .15 [.02, .31] |
|  | stage x suggestion x order | 1 | 37 | 22.79 | <.001 | .38 [.17, .53] |
|  | order | 1 | 37 | 0.03 | .858 | .00 [.00, .03] |

*Note.* SCL – skin conductance level

**Table E:**

*Results of Mixed Analysis of Variance (ANOVA) Models Used to Explain Eye Gaze in N = 36 Participants*

| ANOVA model | Effect | *df_hypothesis_* | *df_error_* | *F* | *p* | _partial_ ƞ^2^ [90% CI] |
| --- | --- | --- | --- | --- | --- | --- |
| *suggestion x CS x order => gaze at AOI1 (cue) during CS* | | |  |  |  |  |
|  | suggestion | 1 | 34 | 9.35 | .004 | .22 [.04, .39] |
|  | suggestion x order | 1 | 34 | 0.42 | .520 | .01 [.00, .13] |
|  | CS | 1 | 34 | 3.43 | .073 | .09 [.00, .26] |
|  | CS x order | 1 | 34 | 1.24 | .273 | .04 [.00, .18] |
|  | suggestion x CS | 1 | 34 | 0.01 | .926 | .00 [.00, .01] |
|  | suggestion x CS x order | 1 | 34 | 0.35 | .558 | .01 [.00, .12] |
|  | order | 1 | 34 | 1.24 | .273 | .04 [.00, .18] |
| *suggestion x CS x order => gaze at AOI2 (face) during CS* | | |  |  |  |  |
|  | suggestion | 1 | 34 | 11.76 | .002 | .26 [.07, .43] |
|  | suggestion x order | 1 | 34 | 0.76 | .391 | .02 [.00, .15] |
|  | CS | 1 | 34 | 11.21 | .002 | .25 [.06, .42] |
|  | CS x order | 1 | 34 | 3.73 | .062 | .10 [.00, .27] |
|  | suggestion x CS | 1 | 34 | 0.16 | .691 | .01 [.00, .10] |
|  | suggestion x CS x order | 1 | 34 | 0.11 | .745 | .00 [.00, .09] |
|  | order | 1 | 34 | 0.03 | .874 | .00 [.00, .03] |
| *suggestion x US x order => gaze at AOI1 (cue) during US* | | |  |  |  |  |
|  | suggestion | 1 | 34 | 14.62 | .001 | .30 [.10, .47] |
|  | suggestion x order | 1 | 34 | 3.26 | .080 | .09 [.00, .25] |
|  | US | 1 | 34 | 8.78 | .006 | .21 [.04, 38] |
|  | US x order | 1 | 34 | 0.74 | .395 | .02 [.00, .15] |
|  | suggestion x US | 1 | 34 | 0.08 | .780 | .00 [.00, .08] |
|  | suggestion x US x order | 1 | 34 | 2.55 | .120 | .07 [.00, .23] |
|  | order | 1 | 34 | 0.40 | .534 | .01 [.00, .12] |
| *suggestion x US x order => gaze at AOI2 (face) during US* | | |  |  |  |  |
|  | suggestion | 1 | 34 | 15.75 | <.001 | .32 [.11, .48] |
|  | suggestion x order | 1 | 34 | 3.29 | .079 | .09 [.00, .25] |
|  | US | 1 | 34 | 18.03 | <.001 | .35 [.14, .51] |
|  | US x order | 1 | 34 | 2.23 | .145 | .06 [.00, .22] |
|  | suggestion x US | 1 | 34 | 0.01 | .942 | .00 [.00, .01] |
|  | suggestion x US x order | 1 | 34 | 2.97 | .094 | .08 [.00, .24] |
|  | order | 1 | 34 | 2.63 | .114 | .07 [.00, .23] |

*Note.* All results involving area of interest (AOI) as a factor are Greenhouse-Geisser corrected. CS – conditioned stimulus; US – unconditioned stimulus.

**Table F:**

*Results of Mixed Analysis of Variance (ANOVA) Models Used to Explain Skin Conductance Response (SCR) and Tonic Skin Conductance Level (SCL), Calculated Separately for Round 1 and 2 of the Paradigm, and Comparison Between Both Rounds (N = 39 Participants)*.

|  |  |  |  | Round 1: | | |  | Round 2: | | |  | Round 1 versus 2: | | |
| --- | --- | --- | --- | --- | --- | --- | --- | --- | --- | --- | --- | --- | --- | --- |
| ANOVA model | Effect | *df_hyp._* | *df_error_* | *F* | *p* | *partial ƞ2* [90% CI] |  | *F* | *p* | *partial ƞ2* [90% CI] |  | *F* | *p* | *partial ƞ2* [90% CI] |
| CS x order => SCR to CS (learning stage) | | | | | | | | | | | | | | |
|  | CS | 1 | 37 | 0.15 | .698 | .00 [.00, .09] |  | 2.44 | .127 | .06 [.00, .21] |  | 0.42 | .520 | .01 [.00, .12] |
|  | CS x order | 1 | 37 | 0.52 | .475 | .01 [.00, .12] |  | 0.24 | .630 | .01 [.00, .10] |  | 0.73 | .397 | .02 [.00, .14] |
|  | order | 1 | 37 | 0.32 | .577 | .01 [.00, .11] |  | 1.10 | .300 | .03 [.00, .16] |  | 1.41 | .243 | .04 [.00, .17] |
| US x order => SCR to US (learning stage) | | | | | | | | | | | | | | |
|  | US | 1 | 37 | 52.56 | <.001 | .59 [.40, .69] |  | 32.09 | <.001 | .46 [.26, .60] |  | 9.81 | .003 | .21 [.05, .38] |
|  | US x order | 1 | 37 | 10.70 | .002 | .22 [.05, .39] |  | 0.26 | .615 | .01 [.00, .10] |  | 4.36 | .044 | .11 [.002, .27] |
|  | order | 1 | 37 | 11.90 | .001 | .24 [.07, .41] |  | 0.09 | .761 | .00 [.00, .08] |  | 5.04 | .031 | .12 [.01, .28] |
| CS x order => SCR to CS (test stage) | | | | | | | | | | | | | | |
|  | CS | 1 | 37 | 10.06 | .003 | .21 [.05, .38] |  | 11.33 | .002 | .23 [.06, .40] |  | 0.39 | .535 | .01 [.00, .11] |
|  | CS x order | 1 | 37 | 1.70 | .200 | .04 [.00, .18] |  | 5.50 | .024 | .13 [.01, .29] |  | 0.09 | .766 | .00 [.00, .08] |
|  | order | 1 | 37 | 2.11 | .155 | .05 [.00, .20] |  | 0.04 | .853 | .00 [.00, .04] |  | 1.40 | .244 | .04 [.00, .17] |
| stage x order => tonic SCL | | | | | | | | | | | | | | |
|  | stage (learning vs. test) | 1 | 37 | 7.11 | .011 # | .16 [.02, .33] |  | 12.50 | .001 § | .25 [.07, .42] |  | 22.79 | <.001 | .38 [.17, .53] |
|  | stage x order | 1 | 37 | 3.65 | .064 | .09 [.00, .25] |  | 1.05 | .313 | .03 [.00, .15] |  | 0.96 | .334 | .03 [.00, .15] |
|  | order | 1 | 37 | 0.59 | .447 | .02 [.00, .13] |  | 0.83 | .370 | .02 [.00, .14] |  | 0.03 | .858 | .00 [.00, .03] |

*Note.* For ANOVA models calculated separately for each round, a significant effect of between-subjects factor “order” indicates different responses in the groups receiving the high versus low affect sharing suggestion in this round.

CS – conditioned stimulus; US – unconditioned stimulus.

#) Learning stage < test stage; §) Learning stage > test stage.

**Table G:**

*Average (Mean +/- SD) Results on Trait Measures in N=39 Participants (30 of them Female).*

|  | Mean | SD |
| --- | --- | --- |
| Age (years) | 19.97 | 1.71 |
| QCAE-C Cognitive Empathy | 62.49 | 6.86 |
| QCAE-A Affective Empathy | 37.89 | 4.84 |
| Osborn Vicarious Pain (no=0 / yes=1) | 0.64 | 0.49 |
| STAI-Y1 State anxiety | 27.55 | 10.05 |
| STAI-Y2 Trait anxiety | 41.95 | 10.25 |
| AQ Autism score | 8.56 | 4.99 |
| HGSHS:A Hypnotizability | 8.77 | 1.31 |

**Table H:**

*Spearman Correlations between SCL, SCR and Eye Gaze observed in the High Affect Sharing Condition in N=36 participants (controlling for order)*

| *High affect sharing:* | Tonic SCL (learning) | Tonic SCL (test) | SCR: UR (learning) | SCR: CR (learning)§ | SCR: CR (test) |
| --- | --- | --- | --- | --- | --- |
| Tonic SCL (learning stage) | 1.00 | ***.42**** | .03 | .10 | -.07 |
| Tonic SCL (test stage) | ***.42**** | 1.00 | -.06 | -.05 | .27 |
| SCR: UR (learning stage) | .03 | -.06 | 1.00 | .10 | ***.33**** |
| SCR: CR (learning stage)§ | .10 | -.05 | .10 | 1.00 | .30 |
| SCR: CR (test stage) | -.07 | .27 | ***.33**** | .30 | 1.00 |
| % gaze at face vs. cue during CS+ | -.09 | ***.44***** | .19 | .02 | .14 |
| % gaze at face vs. cue during CS– | -.01 | ***.45***** | .18 | -.09 | .09 |
| % gaze at face vs. cue during US | -.05 | ***.35**** | .27 | .18 | .30 |
| % gaze at face vs. cue during US absence | -.20 | .28 | -.01 | -.13 | .25 |
| % gaze at face vs. cue during all events # | -0.10 | ***.48***** | 0.18 | 0.00 | 0.19 |

*Note*. Df = 34. SCL – skin conductance level; SCR – skin conductance response; UR – unconditioned response; CR – conditioned response

§) For the correlational analysis, only the last 3 trials of each condition are included in the calculation of the CR in the learning stage, as learning was more likely to have already occurred at this point

#) Gaze times were averaged across all events (i.e., including CS+, CS-, US, US absence events) to represent overall gaze preference

*)p<.05; **) p<.01

**Table I:**

*Spearman Correlations between SCL, SCR and Eye Gaze observed in the Low Affect Sharing Condition in N=36 participants (controlling for order)*

| Low affect sharing: | Tonic SCL (learning) | Tonic SCL (test) | SCR: UR (learning) | SCR: CR (learning)§ | | SCR: CR (test) |
| --- | --- | --- | --- | --- | --- | --- |
| Tonic SCL (learning stage) | 1.00 | -.23 | .27 | .07 | -.14 | |
| Tonic SCL (test stage) | -.23 | 1.00 | .12 | .08 | .15 | |
| SCR: UR (learning stage) | .27 | .12 | 1.00 | .05 | -.11 | |
| SCR: CR (learning stage)§ | .07 | .08 | .05 | 1.00 | .25 | |
| SCR: CR (test stage) | -.14 | .15 | -.11 | .25 | 1.00 | |
| % gaze at face vs. cue during CS+ | .04 | -.01 | .17 | .00 | -.06 | |
| % gaze at face vs. cue during CS– | .11 | -.12 | .09 | -.02 | .00 | |
| % gaze at face vs. cue during US | .10 | .17 | -.01 | ***-.36**** | -.04 | |
| % gaze at face vs. cue during US absence | -.01 | .04 | .02 | -.17 | .02 | |
| % gaze at face vs. cue during all events # | 0.04 | 0.03 | 0.08 | -0.12 | -0.04 | |

Note. Df = 34. SCL – skin conductance level; SCR – skin conductance response; UR – unconditioned response; CR – conditioned response

§) For the correlational analysis, only the last 3 trials of each condition are included in the calculation of the CR in the learning stage, as learning was more likely to have already occurred at this point

#) Gaze times were averaged across all events (i.e., including CS+, CS-, US, US absence events) to represent overall gaze preference

*)p<.05

**Table J:**

*Spearman Correlations among the Differences in SCL, SCR and Eye Gaze Observed between Hypnotically Induced High and Low Affect Sharing in N=36 participants (controlling for order)*

|  | Tonic SCL (learning) | Tonic SCL (test) | SCR: UR (learning) | SCR: CR (learning)§ | SCR: CR (test) |
| --- | --- | --- | --- | --- | --- |
| Tonic SCL (test stage) | ***.48***** | -- | .19 | .18 | .12 |
| SCR: UR (learning stage) | ***.42**** | .19 | -- | .07 | -.03 |
| SCR: CR (learning stage)§ | .11 | .18 | .07 | -- | ***.45***** |
| SCR: CR (test stage) | .00 | .12 | -.03 | ***.45***** | -- |
| % gaze at face vs. cue during CS+ | .26 | .15 | .05 | .19 | -.16 |
| % gaze at face vs. cue during CS– | ***.34**** | -.02 | .03 | .22 | -.13 |
| % gaze at face vs. cue during US | ***.40**** | .26 | -.01 | -.01 | .02 |
| % gaze at face vs. cue during US absence | .17 | .01 | -.33 | .14 | -.02 |
| *% gaze at face vs. cue during all events #* | .32 | .04 | -.07 | .16 | -.08 |

*Note*. Df = 34. SCL – skin conductance level; SCR – skin conductance response; UR – unconditioned response; CR – conditioned response

§) For the correlational analysis, only the last 3 trials of each condition are included in the calculation of the CR in the learning stage, as learning was more likely to have already occurred at this point

#) Gaze times were averaged across all events (i.e., including CS+, CS-, US, US absence events) to represent overall gaze preference

*)p<.05; **)p<.01


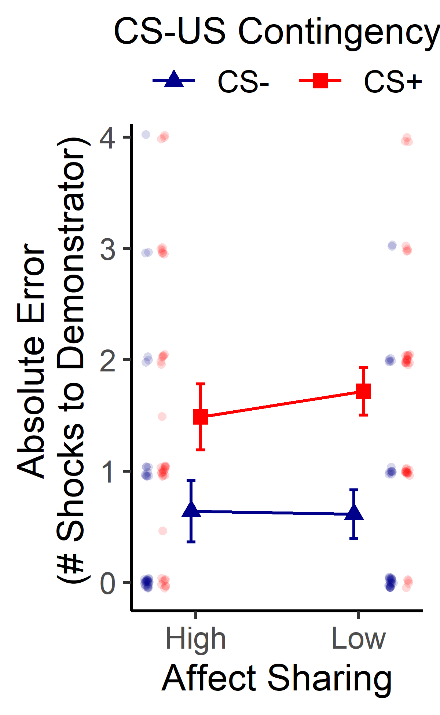


**Figure A**. Results of the post-hypnosis interview showing the effects of hypnotic suggestions for high / low affect sharing on declarative memory of the contingency between CS (colored square) and US (shock / no shock to demonstrator), calculated as the absolute difference between actual and remembered number of shocks delivered to the demonstrator with each CS during the videos. Error bars reflect 95% confidence intervals corrected for within-subject designs (see Methods). CS – conditioned stimulus; US – unconditioned stimulus. Results of individual participants are shown laterally as semi-transparent dots.


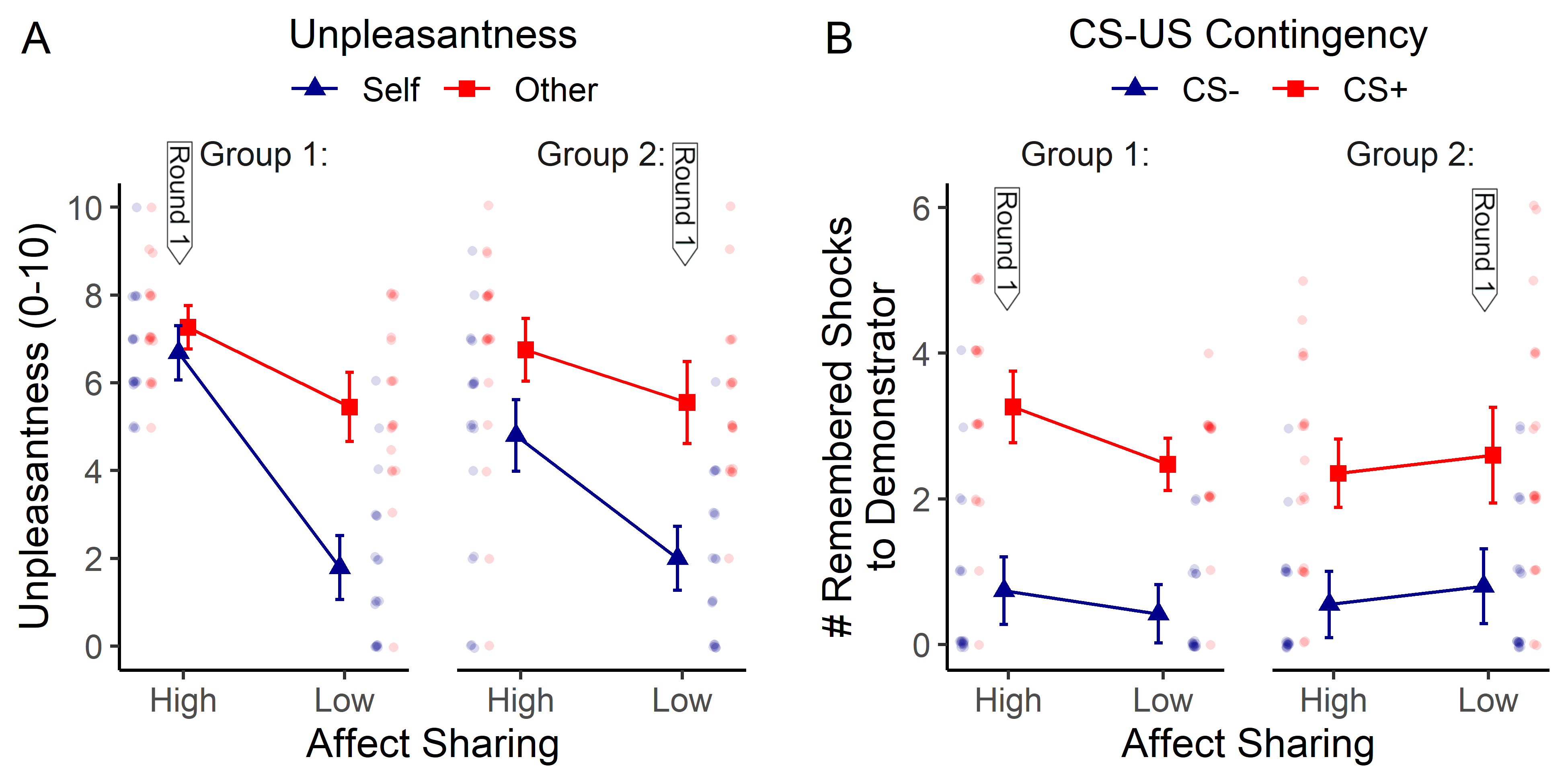


**Figure B:** Results of the post-hypnosis interview showing the effects of hypnotic suggestions for high / low affect sharing on ratings of unpleasantness and declarative memory of the contingency between CS and US, shown separately for the two groups receiving the high affect sharing condition in round 1 (group 1) or in round 2 (group 2). The thick vertical arrows (“Round 1”) indicate which suggestion was delivered first in this group. A) Participants rated how unpleasant the shocks were for the demonstrator in the video (“other”) and how unpleasant it was for themselves to watch this (“self”). B) Participants indicated how many shocks the demonstrator had received following the CS+ and CS-, respectively. Error bars reflect 95% confidence intervals corrected for within-subject designs (see Methods). CS – conditioned stimulus; US – unconditioned stimulus. Results of individual participants are shown laterally as semi-transparent dots.

**
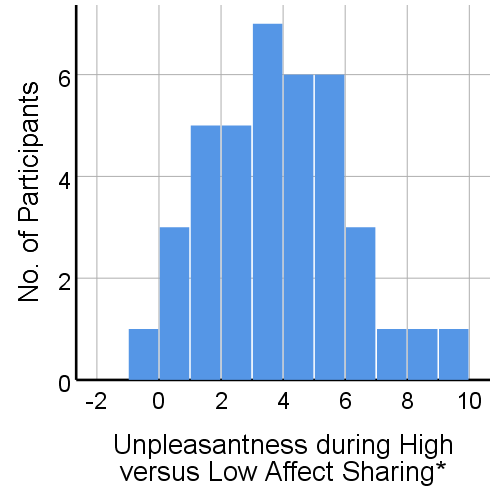
**

**Figure C**: Distribution of the individual participants’ ratings on how unpleasant it was for them to watch the demonstrator receive shocks during high versus low affect sharing

*) residualized of order effects.


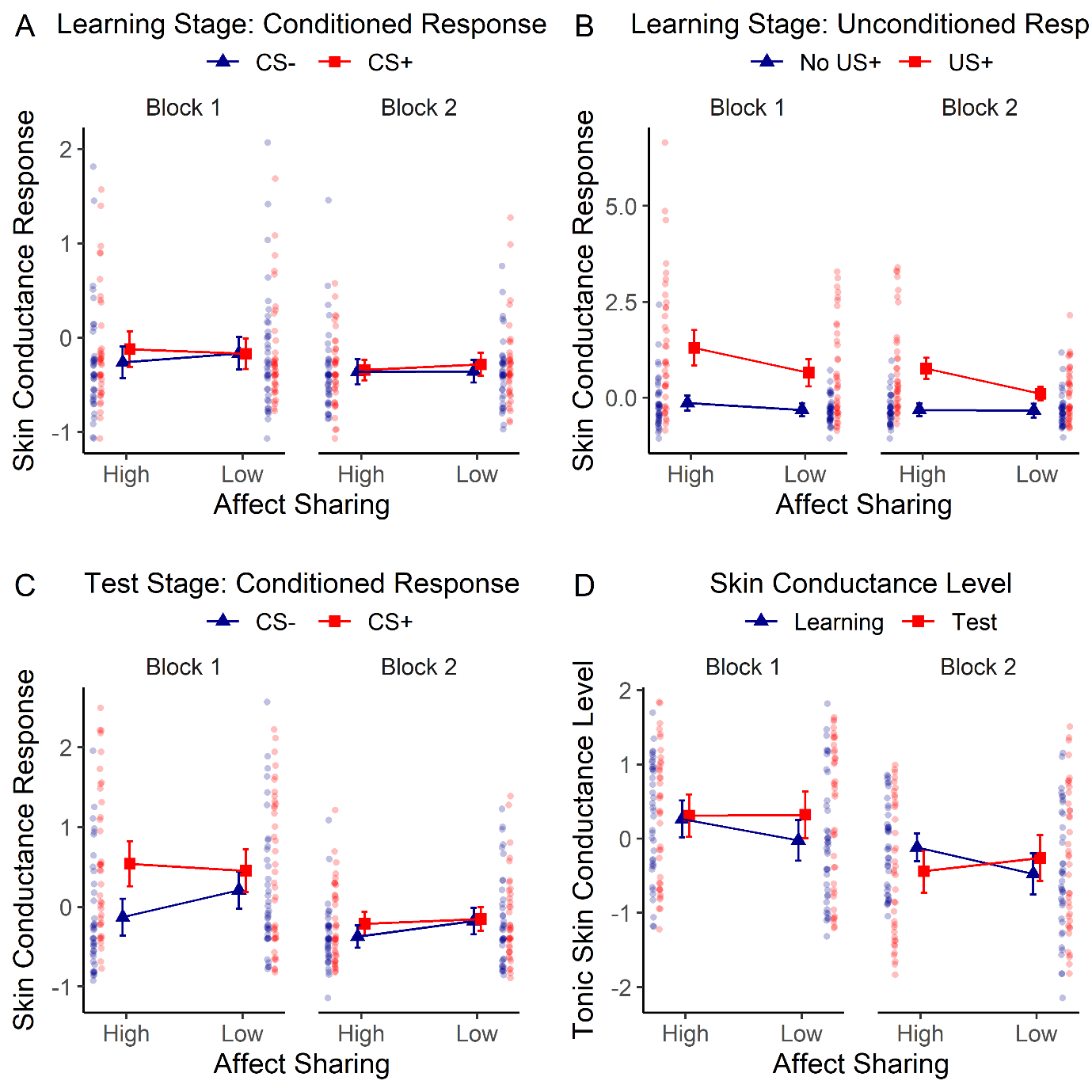


**Figure D**. Effects of hypnotic suggestions for high versus low affect sharing on skin conductance response (SCR) in the learning (A, B) and test stage (C) and on tonic skin conductance level (SCL) across both stages (D), expressed as z-scores. A) SCR to seeing the colored square (CS+ or CS-) in the learning stage, depicted separately for trials 2-3 (block 1) and trials 4-6 (block 2). B) SCR to seeing the demonstrator receive shocks (US) or no shocks (US absence) in the learning stage. The difference in responses to US and US absence indicates the unconditioned response. C) SCR to seeing the colored square (CS+ or CS-) in the test stage. The difference in responses to CS+ and CS- indicates the conditioned response. D) Tonic skin conductance level (SCL) observed in the learning and test stage. Error bars reflect 95% confidence intervals corrected for within-subject designs (see Methods). Results of individual participants are shown laterally as semi-transparent dots.

**
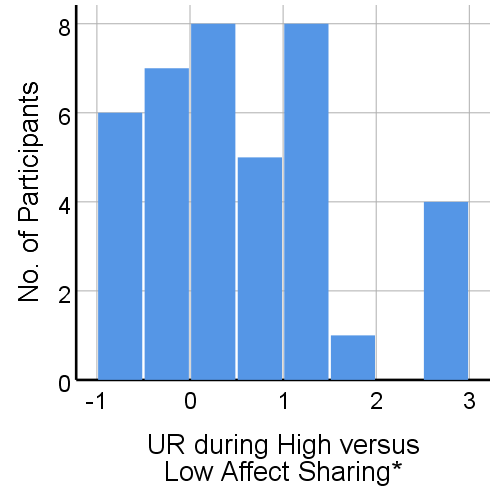
**

**Figure E**: Distribution of the individual participants’ unconditioned response (SCR) to seeing the demonstrator receive shocks during high versus low affect sharing in the learning stage.

*) residualized of order effects.

**Supplementary Discussion of SCR Results in the Test Stage**

As can be seen in Figure D, panel C, the higher conditioned response observed under high versus low affect sharing in the test stage was evident only in the early trials (block 1), not in the later trials (block 2) of the test stage. -The observed pattern of results is more compatible with a true learning effect, rather than slower extinction under high affect sharing in the test stage. Neither was there any difference in tonic sympathetic arousal (skin conductance level) between high and low affect sharing in the test stage, which could have served as an alternative explanation for this effect – see Figure D, panel D and Table D in the Supplementary Material. It seems therefore most plausible to interpret the conditioned response observed in the test stage as the result of learning processes engaged in the vicarious learning stage. This interpretation is further supported by the fact that the conditioned response was correlated between learning and test stage across participants – see Table 1 in the main manuscript.

**References**

Baron-Cohen, S., Wheelwright, S., Skinner, R., Martin, J., & Clubley, E. (2001). The Autism-Spectrum Quotient (AQ): Evidence from Asperger Syndrome/High-Functioning Autism, Malesand Females, Scientists and Mathematicians. *Journal of Autism and Developmental Disorders*, *31*(1), 5–17. https://doi.org/10.1023/A:1005653411471

Georgi, E., Petermann, F., & Schipper, M. (2014). Are empathic abilities learnable? Implications for social neuroscientific research from psychometric assessments. *Social Neuroscience*, *9*(1), 74–81. <https://doi.org/10.1080/17470919.2013.855253>

Grimm, Jürgen (Hg.) (2009): State-Trait-Anxiety Inventory nach Spielberger. Deutsche Lang- und Kurzversion. – Methodenforum der Universität Wien: MF-Working Paper 2009/02

Gulas, C. S., McKeage, K. K., & Weinberger, M. G. (2010). It’s just a joke: Violence against males in humorous advertising. *Journal of Advertising*, *39*(4), 109–120. https://doi.org/10.2753/JOA0091-3367390408

Laux, L., Glanzmann, P., Schaffner, P., & Spielberger, C.D. (1981). Das State-Trait-Angstinventar (Testmappe mit Handanweisung, Fragebogen STAI-G Form X 1 und Fragebogen STAI-G Form X 2); Weinheim: Beltz.

Osborn, J., & Derbyshire, S. W. G. (2010). Pain sensation evoked by observing injury in others: *Pain*, *148*(2), 268–274. <https://doi.org/10.1016/j.pain.2009.11.007>

Zaki, J. (2014). Empathy: A motivated account. *Psychological bulletin, 140*(6), 1608-1647. https://doi.org/10.1037/a0037679
