# Supplementary material for: The causal role of affect sharing in driving vicarious fear learning
Source: PLoS One. 2022 Nov 18;17(11):e0277793. doi: 10.1371/journal.pone.0277793 (PMC9674158; doi:10.1371/journal.pone.0277793)
Supplement: S2 File — (ZIP) [file pone.0277793.s002.zip › S2_File/00analysis_walkthrough.docx]

**Analysis walk-through:**

Important Notes:

- Analysis steps conducted by hand are preceded by the note “*BY HAND*”
- Explanations about the meaning of the variables in the raw data file (“00rawdata.sav”) can be found in the Excel file “00variable_labels.xlsx”.

**(A) Self-report data:**

Do in SPSS 25:

1. Open SPSS data file “00rawdata.sav”
2. Run SPSS script “self01_n39_stats_selfreport.sps” to conduct the statistical analysis of the self-report data

**(B) Skin conductance response (SCR) and skin conductance level (SCL):**

Do in Matlab R2017b:

1. Run Matlab script “SCR00_poly2leda2digi.m” to transform the original output produced by the SCR measurement device (which is in format *.Poly5) into *.mat format compatible with Ledalab.
2. Run Matlab script “SCR01_anl02.m” followed by “SCR02_EDA_Results_anl02.m” to calculate log-transformed amplitudes of SCR occurring in response to each CS (learning and test stage)
3. Run Matlab script “SCR03_anl03.m” followed by “SCR04_EDA_Results_anl03.m” to calculate log-transformed amplitudes of SCR occurring in response to each US (learning stage only)
4. Run Matlab script “SCR10_anl04_SCL.m” followed by “SCR11_EDA_Results_anl04_SCL.m” to calculate log-transformed SCL occurring across the whole duration of each trial (learning and test stage)

Do in SPSS 25:

1. *BY HAND*: Import SCR and SCL data in SPSS
2. Run SPSS script SCR05_anl02_compute_vars_AmpSum_log to do the following for SCR responses to the CS: Correctly name SCR variables, calculate z-transformed (within each participant) SCR variables, and average SCR values across trials 2-3 and across trials 4-6 of each condition (the first trial from each condition is excluded from the analysis).
3. Run SPSS script SCR06_anl03_compute_vars_AmpSum_log to do the following for SCR responses to the US (learning stage only): Correctly name SCR variables, calculate z-transformed (within each participant) SCR variables, and average SCR values across trials 2-3 and across trials 4-6 of each condition (first trial of each condition is excluded from the analysis).
4. *BY HAND*: Combine SCR, SCL and self-report data in a single SPSS data file
5. Run SPSS script “SCR07_n39_stats_SCR.sps” to conduct the statistical analysis of SCR data
6. Run SPSS script “SCR08_stagewise_zscores.sps” to calculate SCR variables z-transformed separately for the learning and for the test stage, to avoid spurious anticorrelations in the correlation analysis
7. Run SPSS script “SCR09_correlate.sps” to create SCR and eye gaze variables partialled from order effects using multiple regression, and calculate Spearman correlations among these variables.
8. Run SPSS script SCR12_anl04_compute_vars_Tonic_log to do the following for SCL during the whole duration of each trial: Correctly name SCL variables, calculate z-transformed (within each participant) SCL variables, and average SCL values across trials 2-6 of each condition (the first trial from each condition is excluded from the analysis).
9. Run SPSS script “SCR13_n39_stats_SCL.sps” to conduct the statistical analysis of SCL data.

**(C) Eye tracking:**

Do in Data Viewer 3.1.97 for each viewing session:

1. *BY HAND* Load EDF files created by the eye tracker recording, change default paths
2. *BY HAND* Load interest area template “eye01_interest_areas_demonstrator1.ias” or “eye02_interest_areas_demonstrator2.ias”, depending on which demonstrator was used for this session
3. *BY HAND* Load report settings template “eye03_report_settings.props” and interest period template “eye04_interest_periods.props”; apply interest areas to all files; define variables (demonstrator, suggestion, round, CS, US, trial, participant) for each trial using the trial variable value editor and the trial variable manager
4. *BY HAND* Save different reports (Trial Report and Aggregate Interest Area Report) for each interest period, changing the variable grouping accordingly, to obtain all relevant output variables.

Do in SPSS 25:

1. *BY HAND* Import Trial Report to SPSS
2. Run SPSS script “eye05_compute_vars_trialreport.sps”
3. *BY HAND* check output and exclude participants with less than 1000ms (IP01) and/or less than 600ms (IP03) total valid fixation time in at least one trial from the sample
4. *BY HAND* Import Aggregate Interest Area Report to SPSS, separately for each interest period
5. Run SPSS script “eye06_compute_vars_AIAreport_IP01.sps” and save SPSS data file
6. Run SPSS script “eye07_compute_vars_AIAreport_IP03.sps” and save SPSS data file
7. *BY HAND* Transpose both data files to have one row per participant and one column per trial, combine to a single data file
8. Run SPSS “eye08_script n36_stats_eyegaze.sps” to conduct the statistical analysis of eye gaze data

**(D) Plots:**

To create line graphs showing confidence intervals for repeated measures designs following Loftus & Masson (2003) using an SPSS macro described in Wright (2007):

Do in SPSS 25 separately for each line plot:

1. *BY HAND* create SPSS data files containing only the data included in the respective line plots
2. Run scripts “plot01…” to “plot02…” to create adjusted self-report data.
3. Run script “plot03…” (applying it to the correct input files) to create plots for self-report data in which confidence intervals adjusted for repeated measures will appear, without changing the condition means.
4. Repeat the last 2 steps for SCR data (“plot04…” to “plot07…”), eyegaze data (“plot08…” to “plot14….”) and SCL data (“plot15…”) to obtain plots for SCR data and eyegaze data.

Do in RStudio:

1. Run script plot16_Fig_D_4_5.Rmd to calculate line plots including individual data points for Figures D (in S1 File), 4 and 5.
2. Run script plot17_Fig_3_B.Rmd to calculate line plots including individual data points for Figures 3 and B (in S1 Fiile).
3. Run script plot18_Fig_A.Rmd to calculate line plots including individual data points for Figure A (in S1 File).
4. Run script plot19_Fig2_noblock.Rmd to calculate line plots including individual data points for Figure 2.
